# Influence of exogenous glucagon‐like peptide 2 on mechanisms of enteric neurotransmitter‐mediated vasorelaxation in the isolated bovine mesenteric artery and vein

**DOI:** 10.14814/phy2.71134

**Published:** 2026-10-09

**Authors:** Gabriel S. S. David, Erica C. Maples, James L. Klotz, Ronald J. Trotta

**Affiliations:** ^1^ Department of Animal and Food Sciences University of Kentucky Lexington Kentucky USA; ^2^ Forage‐Animal Production Research Unit United States Department of Agriculture—Agricultural Research Service Lexington Kentucky USA

**Keywords:** acetylcholine, cattle, myography, serotonin, small intestine, vasoactive intestinal peptide

## Abstract

Exogenous glucagon‐like peptide 2 (GLP‐2) administration increases small intestinal growth and mesenteric blood flow, although the vascular mechanisms involved remain unclear. The objective of this study was to characterize the effects of exogenous GLP‐2 administration on glucagon‐like peptide 2 (GLP‐2)‐, vasoactive intestinal peptide (VIP)‐, serotonin (5‐HT)‐, and acetylcholine (ACh)‐mediated vascular relaxation in isolated bovine mesenteric arteries (MA) and mesenteric veins (MV). Ten steers received repeated subcutaneous injections of bovine serum albumin or GLP‐2 for 48 h before vessel collection. Vascular dimensions, vasoactive responses using myography, and mRNA expression of receptors associated with vasorelaxation were evaluated. Exogenous GLP‐2 did not directly induce vasorelaxation in isolated MA or MV despite detectable *GLP2R* mRNA expression in both vessels, suggesting that GLP‐2‐mediated increases in mesenteric blood flow occur through indirect mechanisms. Vasoactive intestinal peptide induced dose‐dependent vasorelaxation in both vessel types, and only *VIPR2* mRNA was detected. Serotonin‐mediated responses were not altered by exogenous GLP‐2 treatment. In contrast, exogenous GLP‐2 enhanced ACh‐mediated vasorelaxation in the MA at greater agonist concentrations and increased *NOS3* mRNA expression in the MV. These findings suggest that cholinergic signaling pathways may contribute to GLP‐2‐associated vasoregulation in the bovine MA.

## 1 INTRODUCTION

Glucagon‐like peptide 2 (GLP‐2) is an endogenously secreted gastrointestinal hormone that plays a central role in stimulating intestinal growth and function. Exogenous GLP‐2 administration increases small intestinal mass, particularly within the jejunum, through structural remodeling of the intestinal epithelium, including increased villus height and crypt depth by increasing cellular proliferation and decreasing cell apoptosis and proteolysis across multiple species (Brubaker et al., 1997; Burrin et al., 2000, 2005; Drucker et al., 1996; Taylor‐Edwards et al., 2011; Tsai, Hill, Asa, et al., 1997; Tsai, Hill, & Drucker, 1997). Intestinotrophic effects of GLP‐2 are mediated through downstream activation of insulin‐like growth factor‐1 and epidermal growth factor signaling, which subsequently stimulate Wnt/β‐catenin and PI3K/Akt pathways involved in cellular proliferation and differentiation, while suppressing ubiquitin‐mediated proteolysis (Austin et al., 2015; Trotta, Swanson, et al., 2024; Yusta et al., 2009).

The intestinal growth response to exogenous GLP‐2 is accompanied by marked alterations in splanchnic hemodynamics, including increased blood flow and decreased vascular resistance within the superior mesenteric artery and portal vein in both ruminant and nonruminant species (Deniz et al., 2007; Guan et al., 2003, 2006; Stephens et al., 2006; Taylor‐Edwards et al., 2011). However, the mechanisms regulating GLP‐2‐mediated vascular responses remain incompletely understood and may differ across species. In pigs, GLP‐2‐induced increases in mesenteric blood flow were nitric oxide (NO)‐dependent (Guan et al., 2003), potentially through stimulation of endothelial NO synthase activity and subsequent NO‐mediated vasodilation of mucosal capillaries (Koopmann et al., 2008). In addition, the GLP‐2 receptor is co‐expressed with vasoactive intestinal peptide (VIP)‐ and serotonin (5‐HT)‐containing enteric neurons (Guan et al., 2006), suggesting that enteric neurotransmitters may be involved in downstream vasodilatory signaling pathways. In support, exogenous GLP‐2 administration increased serum 5‐HT concentrations in mice (Iwai et al., 2013), potentially secondary to increased small intestinal growth and proliferation, resulting in an indirect increase in intestinal 5‐HT synthesis. In contrast, recent work in rats demonstrated that the increase in mesenteric blood flow elicited by GLP‐2 occurred independently of NO and VIP signaling pathways, indicating the presence of alternative downstream mediators that remain undefined (Galsgaard et al., 2025). Collectively, these findings suggest that GLP‐2‐mediated regulation of mesenteric blood flow could be multifactorial and species‐dependent.

Despite evidence that exogenous GLP‐2 alters mesenteric hemodynamics in cattle (Taylor‐Edwards et al., 2011), the neurovascular mechanisms mediating these responses remain poorly characterized in ruminants. Taylor‐Edwards et al. (2011) reported that exogenous GLP‐2 administration increased blood flow to splanchnic tissues in cattle, with acute exposure (1 day) producing a greater increase in superior mesenteric arterial blood flow than prolonged exposure (10 days). These findings suggest that vascular responsiveness to exogenous GLP‐2 may adapt over time and may involve both direct and indirect neurovascular signaling pathways. However, the contribution of enteric neurotransmitter‐mediated vasorelaxation to GLP‐2‐induced vascular responses has not been completely described. Therefore, we hypothesized that in vivo GLP‐2 administration would alter subsequent ex vivo mesenteric vascular relaxation responses to GLP‐2 and enteric neurotransmitters, including VIP, 5‐HT, and acetylcholine (ACh). The objectives of this study were to characterize the effects of exogenous GLP‐2 administration on mesenteric vascular morphology, vasorelaxation responses, and the mRNA expression of genes associated with receptor‐mediated vasorelaxation in bovine jejunal mesenteric arteries and veins.

## 2 MATERIALS AND METHODS

### 2.1 Glucagon‐like peptide 2 preparation and administration

Glucagon‐like peptide 2 was synthesized (Alan Scientific Inc., Gaithersburg, MD) based on the native bovine sequence (H‐HADGSFSDEMNTVLDSLATRDFINWLLQTKITD‐OH) (Burrin et al., 2003; Lopez et al., 1983). Peptide purity (96%) was quantified by the manufacturer using high‐performance liquid chromatography. The GLP‐2 solution was prepared to a final concentration of 5 mg/mL by dissolving GLP‐2 in 5 g/L bovine serum albumin (BSA; BP9706100; Fisher Scientific Inc.) in 9 g/L NaCl solution. Aliquots were frozen in 10‐mL screw‐cap tubes at −20°C until use.

### 2.2 Animals and experimental design

Ten Angus × Holstein steers [initial body weight (BW) = 467 ± 106 kg] were randomly assigned to one of two treatment groups (BSA or GLP‐2; *n* = 5 per treatment) in a completely randomized design, with each steer serving as the experimental unit. Sample size was determined using bovine mesenteric artery and vein contractile responses to increasing concentrations of 5‐HT (Trotta et al., 2018; Trotta, Harmon, et al., 2023) using methods described by Berndtson (1991). Steers were housed in individual pens (3 × 3 m) with controlled temperature (20°C) and light (16 h light: 8 h dark) conditions in the Intensive Research Building at the University of Kentucky C. Oran Little Research Center in Versailles, KY, USA. Steers were limit‐fed a corn silage‐based diet once daily to provide two times the net energy required for maintenance. Steers received subcutaneous injections in 12‐h intervals (0, 12, 24, 36, and 48 h) of either 0.5% BSA as a vehicle or 50 μg GLP‐2/kg of BW per injection for 48 h. The 48‐h treatment period was chosen because: (1) increased small intestinal growth occurs after 48 h of exogenous GLP‐2 administration (Burrin et al., 2007), (2) acute exposure (24 h) to exogenous GLP‐2 results in 30% greater superior mesenteric arterial blood flow compared with chronic exposure (240 h) to exogenous GLP‐2 (Taylor‐Edwards et al., 2011), and (3) the magnitude of the plasma GLP‐2 concentration response to exogenous GLP‐2 administration does not differ between acute (24 h) and chronic (240 h) length of exposure (Taylor‐Edwards et al., 2011). Intestinotrophic effects of GLP‐2 are greater when GLP‐2 was administered subcutaneously compared with intramuscular or intraperitoneal injections (Tsai, Hill, Asa, et al., 1997). Therefore, treatments were administered subcutaneously in the neck region anterior to the shoulder. Injection sites were alternated between the left and right sides for morning and evening injections, respectively. Following the final subcutaneous injection at 48 h, steers were transported to the University of Kentucky Meats Laboratory in Lexington, KY, USA. Steers were stunned by penetrating captive bolt and slaughtered by exsanguination.

### 2.3 Blood vessel collection

Approximately 2 h after the final GLP‐2 or BSA injection, jejunal mesenteric arteries (MA) and mesenteric veins (MV) were obtained from the gastrointestinal tracts of the 10 steers (1 steer slaughtered per day) at the University of Kentucky Meats Laboratory. The ileocecal fold of the small intestine was identified to locate the ileal flange and properly orient the gastrointestinal tract for jejunal MA and MV collection. The collateral branch of the cranial MA was located in the jejunal mesentery along the last centrifugal gyrus of the spiral colon (Budras et al., 2011). The point where the collateral branch of the MA splits off from the cranial MA was located and used as the main landmark for sampling distal branches of MA and MV that supply and drain the jejunum. Four consecutive branches were isolated from the jejunum using methodology adapted from Klotz and Barnes (2014). Isolated vessel branches were immersed in oxygenated Krebs–Henseleit buffer (95% O_2_/ 5% CO_2_; pH = 7.4; 11.1 mM D‐glucose; 1.2 mM MgSO_4_; 1.2 mM KH_2_PO_4_; 4.7 mM KCl; 118.1 mM NaCl; 3.4 mM CaCl_2_; 24.9 mM NaHCO_3_; Sigma Chemical Co., St. Louis, MO, USA), stored on ice, and transported to the USDA‐ARS Forage‐Animal Production Research Unit lab.

### 2.4 Blood vessel dissection and vascular dimensions

Blood vessels were dissected and surrounding adipose and connective tissues were removed. One isolated branch of the MA and MV was snap‐frozen in liquid nitrogen and stored at −80°C to preserve RNA. Isolated vessels were sliced into approximately 2‐mm cross‐sections using an adjustable acrylic tissue matrix (Braintree Scientific Inc., Braintree, MA, USA). Using a dissection microscope (Stemi 2000‐C, Carl Zeiss Inc., Oberkochen, Germany) at 12.5× magnification, vascular dimensions of vessel cross‐sections were recorded using Axiovision (version 20, Carl Zeiss Inc.). Vessel wall thickness was calculated as the difference between the outer diameter and inner diameter divided by 2. In addition, vessel cross‐sections were inspected under magnification for abnormalities (structural damage incurred during dissection and cleaning and branches), and abnormal sections were discarded and replaced with viable sections.

### 2.5 Mesenteric artery and vein mRNA expression

Isolated MA and MV (*n* = 4 steers per treatment) were weighed (67.9 ± 17.0 mg), placed into tubes containing 2 mL of TRI reagent (R2050‐1‐50; Zymo Research Inc.), and homogenized (TH115; Omni International Inc.). Homogenates were centrifuged (12,000 × *g*; 5 min; 4°C) and the supernatants were transferred to 1.5 mL nuclease‐free microcentrifuge tubes. Total RNA was extracted using the Direct‐zol RNA mini prep kit (R2051; Zymo Research Inc.), and genomic DNA was eliminated using a Zymo‐Spin IIICG column (C1006‐50‐G; Zymo Research Inc.) and DNase I treatment (E1010; Zymo Research Inc.). Total RNA concentration (156 ± 84.0 ng/μL) was measured using a microvolume UV–VIS spectrophotometer (NanoDrop One; ThermoFisher Scientific Inc.) and RNA purity was assessed by measuring the 260/280 nm ratio (2.00 ± 0.0719) and 260/230 nm ratio (1.90 ± 0.187). The RNA integrity (IQ = 7.96 ± 0.627) was measured using the Qubit RNA Integrity and Quality Assay Kit (Q33221; ThermoFisher Scientific, Inc.) with a Qubit fluorometer (version 4.0). Complementary DNA was synthesized from 750 ng of RNA from each sample using the iScript cDNA Synthesis Kit (1708890; Bio‐Rad Laboratories, Inc.) containing 1 μL of modified Moloney murine leukemia virus reverse transcriptase (1708890; iScript Reverse Transcriptase; Bio‐Rad Laboratories, Inc.) per 20‐μL reaction. The reaction protocol was primed at 25°C for 5 min, reverse transcribed at 46°C for 20 min, enzyme inactivated at 95°C for 1 min, and then cooled to 4°C using a MiniAmp Thermal Cycler (A37028; Applied Biosystems).

Systematic and common gene names, reference sequences, forward and reverse primer sequences, and primer efficiencies are reported in Table 1. Target genes included cholinergic receptor muscarinic 3 (*CHRM3*), glucagon‐like peptide 2 receptor (*GLP2R*), 5‐hydroxytryptamine receptor 4 (*HTR4*), 5‐hydroxytryptamine receptor 7 (*HTR7*), nitric oxide synthase 3 (*NOS3*), vascular endothelial growth factor A (*VEGFA*), vasoactive intestinal peptide receptor 1 (*VIPR1*), and vasoactive intestinal peptide receptor 2 (*VIPR2*). Forward and reverse primers for target and reference genes were designed using Primer3 software to produce one ~150 bp amplicon bridging an exon‐exon junction with an optimal melting temperature of 60°C. Amplification efficiency (95%–115%) was assessed for each primer set by generating a six‐point serial dilution standard curve to span three orders of magnitude. Complementary DNA was amplified using a CFX Opus 96 Real‐Time PCR System (Bio‐Rad Laboratories, Inc.) using a 10‐μL reaction volume [5 μL iTaq Universal Syber Green Supermix (1725120; Bio‐Rad Laboratories, Inc.), 4 μL of diluted cDNA in nuclease‐free water, and 1 μL of primers at 500 nM] with 40 oscillating cycles of denaturing (95°C for 5 s) and annealing/extension (60°C for 30 s). Melt curve analysis was conducted by increasing temperature from 65°C to 95°C at 0.5°C increments with 5 s per increment. A single melt curve peak was observed for each individual well. Multiple reference genes were evaluated for the stability of expression across treatments and vascular tissues: β‐actin (*ACTB*), glyceraldehyde‐3‐phosphate dehydrogenase (*GAPDH*), and succinate dehydrogenase complex flavoprotein subunit A (*SDHA*). Stability was assessed by geNorm analysis (Vandesompele et al., 2002) using qbase+ software (Biogazelle, Zwijnarrde, Belgium). The geometric mean of *ACTB*, *GAPDH*, and *SDHA* had the lowest *M*‐value (*M* < 0.05) and was therefore selected for target gene normalization. Relative mRNA expression was calculated using the 2^−ΔΔCt^ method (Livak & Schmittgen, 2001).

**TABLE 1:** Systematic gene names, symbols, accession numbers, forward and reverse primer sequences, amplicon size, and amplification efficiency of target and reference genes.

Systematic name	Symbol	NCBI accession	Forward sequence	Reverse sequence	Amplicon (bp)	Efficiency (%)
Actin beta	*ACTB*	NM_173979	GCCAGAAGGACTCGTACGTG	AGCTCGTTGTAGAAGGTGTGG	139	107.7
Glyceraldehyde‐3‐phosphate dehydrogenase	*GAPDH*	NM_001034034	AGTTCAACGGCACAGTCAAG	CATCACCCCACTTGATGTTGG	105	102.1
Succinate dehydrogenase complex flavoprotein subunit A	*SDHA*	NM_174178	ATTACGGCATGCCCTTTAGC	TTCCCGAACTTGAGGCTCTG	82	108.3
Cholinergic receptor muscarinic 3	*CHRM3*	NM_174270	ACGAACAACAAAGCGAGCTG	TGAGGAACTGGATGAAGCACTC	143	112.0
Glucagon‐like peptide 2 receptor	*GLP2R*	XM_005220435	AAGTTGAACACCGCACCTTG	ACGTGTGCAGTGGAGTTTTC	128	107.6
5‐hydroxytryptamine receptor 4	*HTR4*	NM_001040485	TTGTGTCTCTTGCCTTTGCG	TGTTGTGAGCAGGACATCCAG	140	110.0
5‐hydroxytryptamine receptor 7	*HTR7*	XM_059881983	TCCAACTACCTGATCGTGTCC	CATGACGTCCATGGCGATAAAG	147	114.0
Nitric oxide synthase 3	*NOS3*	NM_181037	TTTAAGGAAGTGGCCAACGC	TGGTTGCTTTCACTCGCTTG	79	104.5
Vascular endothelial growth factor A	*VEGFA*	NM_174216	GCCTCCGAAACCATGAACTTTC	CCATGAACTTCACCACTTCGTG	142	111.9
Vasoactive intestinal peptide receptor 1	*VIPR1*	NM_001081607.2	CGCATCATTGGAATCCTGGTTC	TGATGTAGTGCACGCCAAAC	127	ND^a^
Vasoactive intestinal peptide receptor 2	*VIPR2*	NM_001206781	AGAAGTTGACGTCCCCAGATG	AGATGCGGATGGGAAAGACAG	133	105.8

^a^ Not determined.

Products of the qPCR reactions were purified using ethanol precipitation. Ten microliters of the purified double‐stranded DNA products were electrophoresed (3% agarose gels; 110 V; 40 min) along with a GeneRuler Low Range DNA Ladder (SM1192; ThermoFisher Scientific Inc.). The gel was stained (S33102; SYBR Safe DNA Gel Stain; ThermoFisher Scientific Inc.) and imaged using ultraviolet light at 302 nm with auto‐optimal settings (Azure Biosystems c600 System). Purified dsDNA products were sequenced by Plasmidsaurus using a GridION (Oxford Nanopore Technologies) and specificity of primer sets for target genes was confirmed.

### 2.6 Standard preparations

Stock solutions (w/v) of endothelin‐1 trifluoroacetate salt (24127; Cayman Chemical), GLP‐2 (Alan Scientific Inc.), VIP (1911; Tocris Bioscience), 5‐HT hydrochloride (H9523; Sigma‐Aldrich), and acetylcholine chloride (A6625; Sigma Aldrich) were prepared in dimethyl sulfoxide (DMSO; 472301; Sigma‐Aldrich). Serial dilutions of agonist stock solutions were prepared daily to achieve desired working concentrations, ranging from 5 × 10^−9^ M to 1 × 10^−5^ M for GLP‐2, 5 × 10^−10^ M to 1 × 10^−6^ for VIP, 5 × 10^−9^ to 1 × 10^−4^ for 5‐HT, and 5 × 10^−7^ M to 1 × 10^−3^ M for ACh. Diluted standards were added to the incubation buffer in 25‐μL aliquots to maintain the solvent (DMSO) amount below 0.5% of the total volume and to achieve the working concentrations in myograph chambers.

### 2.7 Myography

Four multi‐myograph systems (DMT 610 M, Danish Myo Technology, Atlanta, GA) containing four individual chambers per myograph were used for each experiment. Prior to calibration, each myograph was turned on to reach the desired temperature setting (37.5°C). Each chamber of the myograph was calibrated prior to the initiation of the experiment according to the manufacturer's instructions. The blood vessels were mounted onto luminal pins (Wenceslau et al., 2021) and submerged with 5 mL of Krebs–Henseleit buffer and constant gassing (95% O_2_/5% CO_2_; pH = 7.4; 37°C). The Krebs–Henseleit buffer used in myograph chambers was the same composition as the transport buffer with 3 × 10^−5^ M desipramine (D3900; Sigma‐Aldrich) added to inactivate neuronal catecholamine reuptake. Blood vessels were equilibrated with buffer changes every 15 min to allow blood vessels to reach a resting tension of approximately 1 g. Mesenteric artery and vein equilibrations were conducted for 60 min and 90 min, respectively. At the completion of the equilibration period, 25 μL of 2 × 10^−8^ M endothelin‐1 was added to each myograph chamber to pre‐contract each vessel segment for 15 min. Following the endothelin‐1 pre‐contraction, 25 μL of GLP‐2, VIP, 5‐HT, or ACh was added to myograph chambers non‐cumulatively for 5 min in duplicate for each working concentration. Within steer, each agonist was evaluated with a fresh preparation of blood vessels.

### 2.8 Vasorelaxation response and potency measurements

Vasorelaxation responses to treatments were measured for each animal in duplicate and averaged. Isometric changes in tension (measured in millinewtons) of blood vessel segments in response to endothelin‐1, GLP‐2, VIP, 5‐HT, and ACh were digitized and converted to mass units (in grams) using PowerLab16/35 and Chart software (version 8.1, ADInstruments, Colorado Springs, CO). The tension (measured in grams) during the endothelin‐1 pre‐contraction period was determined after plateau (15 min) and corrected for baseline tension (Wenceslau et al., 2021). During the 5‐min treatment incubation period following the pre‐contraction period, the minimum grams of tension were measured. Recorded grams of tension during the 5‐min treatment incubation period were normalized as a percentage of the maximum grams of tension induced by the endothelin‐1 pre‐contraction at plateau to compensate for differences in responsiveness due to vessel size. Vessel vasorelaxation response data were reported as the percent mean vasorelaxation response (± standard error of the mean) of the maximum contractile response produced by the endothelin‐1 pre‐contraction. Vasorelaxation response data were plotted using nonlinear regression with fixed slope (GraphPad Prism 10.4; Dotmatics, Boston, MA) and calculated using a log(agonist) versus response three parameter equation (Wenceslau et al., 2021):

$$y=\text{bottom}+\frac{\left(\mathrm{top}-\text{bottom}\right)}{\left[1+10^{\left(x-\text{logEC}50\right)}\right]},$$

where *y* represents vasoactive response, *x* represents agonist concentration, top and bottom are plateaus in units of the *y*‐axis (percentage of endothelin‐1 pre‐contraction maximum), and 50% effective concentration (EC_50_) is the molar concentration of the agonist producing 50% of the response between plateaus.

### 2.9 Statistical analysis

All data were analyzed for normality using the Shapiro–Wilk test in the UNIVARIATE procedure of SAS. The myography data were analyzed using the MIXED procedure of SAS as a split‐plot design as eight separate experiments (GLP‐2, VIP, 5‐HT, ACh dose–response for MA and for MV). The whole plot factor was the in vivo treatment (s.c. injection): BSA vs. GLP‐2. The sub plot factor was the ex vivo agonist concentration in the myograph. The model includes fixed effects of s.c. injection, agonist concentration, and s.c. injection × agonist concentration their interactions and the whole plot error term was specified as a random effect of animal × s.c. injection interaction. One sample in each treatment did not yield sufficient total RNA to synthesize cDNA for qPCR analysis of all target genes, and thus, *n* = 4 per treatment for relative mRNA expression analyses. Vascular dimensions and relative mRNA expression data were analyzed using the MIXED procedure of SAS for fixed effect of treatment. Non‐normally distributed data were log_2_ transformed prior to analysis of variance and least squares means were back‐transformed for presentation in the table. Results were considered statistically significant at *p* ≤ 0.05.

## 3 RESULTS

### 3.1 Blood vessel dimensions and expression of genes involved in receptor‐mediated vasorelaxation for bovine mesenteric arteries and veins

Glucagon‐like peptide 2 administration did not affect (*p* ≥ 0.23) vessel dimensions of the isolated MA and MV (Table 2). Moreover, in vivo administration of GLP‐2 did not influence the mRNA expression of genes (*CHRM3*, *GLP2R*, *HTR4*, *HTR7*, *VEGFA*, and *VIPR2*) involved in receptor‐mediated vasorelaxation in bovine MA and MV (*p* ≥ 0.41), except for *NOS3* in MV (*p* < 0.03), with greater expression for steers that were treated with exogenous GLP‐2 in vivo (Table 3). The primer efficiencies (Table 1) of *VIPR1* could not be determined because of high *C*_q_ values (>35) in bovine mesenteric blood vessels, indicating a lack of transcript expression (data not shown). Ribonucleic acid extracted from bovine duodenal epithelium was used as a positive control and confirmed valid amplification efficiency (102.7%) and specificity for *VIPR1* (data not shown).

**TABLE 2:** Least squares means for the effect of exogenous GLP‐2 administration on vascular dimensions for bovine mesenteric arteries and veins.

Vessel dimension	Treatment		SEM^a^	*p*‐value
	BSA	GLP‐2		
Mesenteric artery				
Length, mm	1.89	1.93	0.109	0.82
Outer diameter, mm	2.32	2.37	0.168	0.85
Inner diameter, mm	1.65	1.73	0.052	0.31
Vessel wall thickness, mm	0.697	0.733	0.042	0.57
Mesenteric vein				
Length, mm	2.00	1.94	0.082	0.62
Outer diameter, mm	2.65	2.68	0.130	0.87
Inner diameter, mm	1.37	1.32	0.122	0.78
Vessel wall thickness, mm	0.781	0.710	0.037	0.23

^a^ Standard error of the mean (*n* = 5).

**TABLE 3:** Least squares means for the effect of exogenous GLP‐2 administration on mRNA expression of genes involved in receptor‐mediated vasorelaxation for bovine mesenteric arteries and veins.

	Treatment		log_2_(SEM)^a^	*p*‐value
	BSA	GLP‐2		
Mesenteric artery				
*CHRM3*	1.03	2.60	1.621	0.58
*GLP2R*	1.00	1.59	1.467	0.76
*HTR4*	1.00	3.24	1.536	0.46
*HTR7*	1.00	1.65	1.348	0.72
*NOS3*	1.00	0.59	0.613	0.44
*VEGFA*	1.00	1.28	0.518	0.65
*VIPR2*	1.00	1.54	1.212	0.73
Mesenteric vein				
*CHRM3*	1.00	0.37	1.146	0.41
*GLP2R*	1.00	1.09	1.289	0.95
*HTR4*	1.00	0.59	1.069	0.63
*HTR7*	1.00	0.73	1.231	0.71
*NOS3*	1.00	7.28	0.653	0.02
*VEGFA*	1.00	1.00	0.332	0.99
*VIPR2*	1.00	0.68	1.076	0.73

^a^ The log_2_ (standard error of the mean) is reported (*n* = 4). Least squares means were log_2_ transformed for statistical analyses and then back‐transformed for presentation in the table.

### 3.2 Response of the isolated MA to exogenous GLP‐2 and mediation through GLP‐2, VIP, 5‐HT, or ACh

Exogenous GLP‐2 administration did not influence (*p* = 0.35) the vasoactive response of the isolated MA to GLP‐2 (Figure 1a). Increasing concentration of VIP dose‐dependently decreased (*p* < 0.001) the vasoactive response of the isolated MA below the ET‐1 maximum (i.e., vasorelaxation) (Figure 1b). However, exogenous GLP‐2 administration (*p* = 0.55) did not influence the vasoactive response of the isolated MA to VIP. Exogenous GLP‐2 administration did not influence (*p* = 0.37) the vasoactive response of the isolated MA to 5‐HT (Figure 1c). There was an in vivo s.c. injection × ex vivo ACh concentration interaction (*p* < 0.001) in the isolated MA (Figure 1d). As ACh concentration increased, vasorelaxation increased in both treatments; however, at greater ACh concentrations, there was greater vasorelaxation (*p* = 0.03) in GLP‐2‐treated MA compared with the MA from BSA‐treated steers. No differences were observed in ‐log EC_50_ values among treatments for any agonist (GLP‐2, VIP, 5‐HT, and ACh) in the MA (*p* ≥ 0.33; Table 4).

**FIGURE 1:**
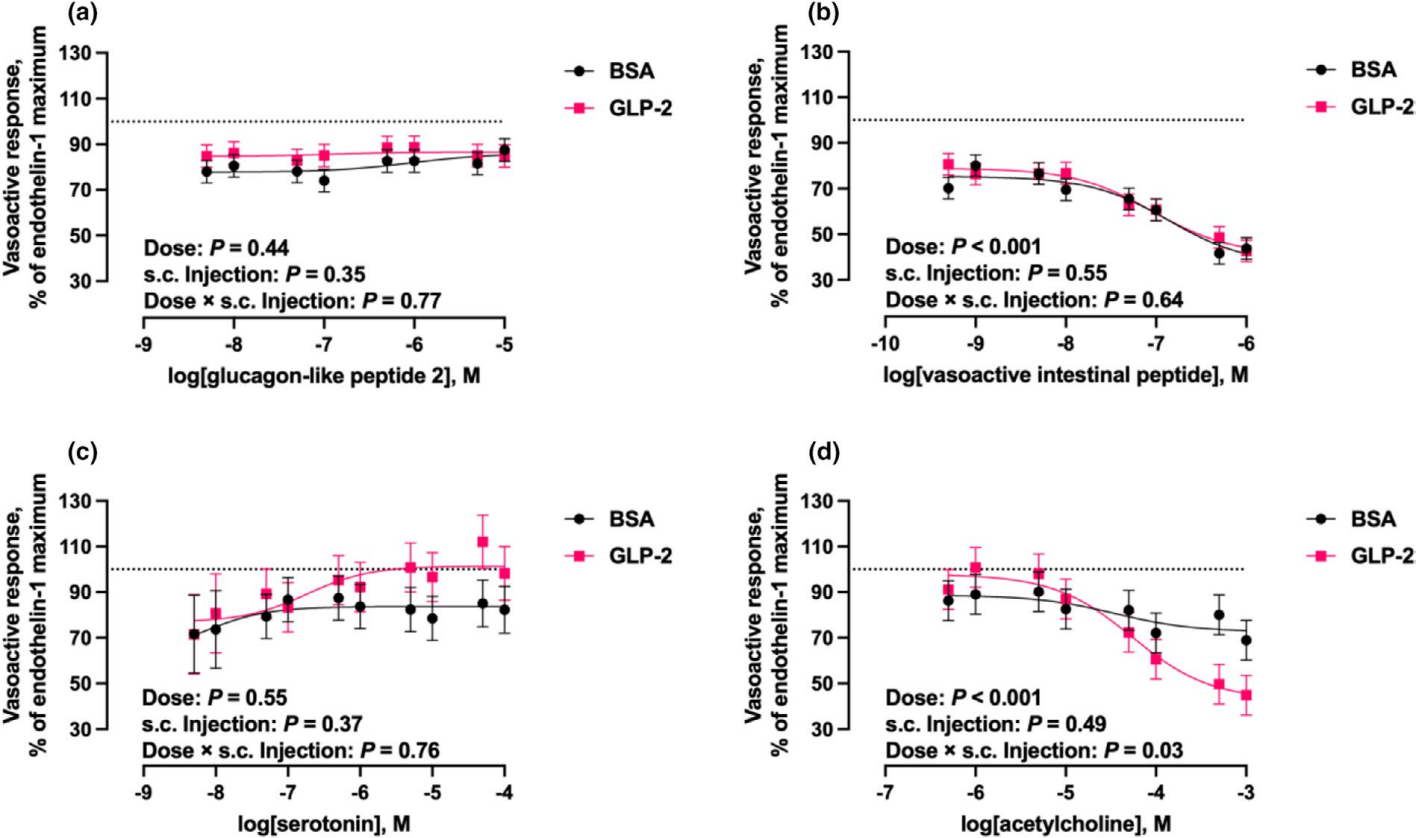
Least squares mean contractile responses (± SEM; *n* = 5) of the mesenteric artery to increasing concentrations of (a) GLP‐2, (b) VIP, (c) 5‐HT, and (d) ACh in steers treated in vivo with bovine serum albumin (BSA; control) or GLP‐2. *p* values: (a) GLP‐2 dose (*p* = 0.44), s.c. injection (*p* = 0.35), and the dose × s.c. injection interaction (*p* = 0.77). (b) VIP dose (*p* < 0.001), s.c. injection (*p* = 0.55), and the dose × s.c. injection interaction (*p* = 0.64). (c) 5‐HT dose (*p* = 0.55), s.c. injection (*p* = 0.37), and the dose × s.c. injection interaction (*p* = 0.76). (d) ACh dose (*p* < 0.001), s.c. injection (*p* = 0.49), and the dose × s.c. injection interaction (*p* < 0.04). The dotted line at 100% represents the maximal contractile response to endothelin‐1.

**TABLE 4:** Least squares means for the effect of exogenous GLP‐2 administration on ‐log EC_50_ values in response to increasing doses of GLP‐2, VIP, 5‐HT, or A.

Agonist	Treatment		SEM^a^	*p*‐value
	BSA	GLP‐2		
Mesenteric artery				
Glucagon‐like peptide 2	6.83	6.86	1.366	0.98
Vasoactive intestinal peptide	7.00	7.27	0.312	0.56
Serotonin	5.83	6.49	0.612	0.47
Acetylcholine	5.73	4.30	0.973	0.33
Mesenteric vein				
Glucagon‐like peptide 2	6.77	5.36	1.140	0.41
Vasoactive intestinal peptide	7.36	9.06	1.122	0.32
Serotonin	6.50	5.26	1.707	0.62
Acetylcholine	2.77	4.18	0.509	0.09

^a^ Standard error of the mean (*n* = 5).

### 3.3 Response of the isolated MV to exogenous GLP‐2 and mediation through GLP‐2, VIP, 5‐HT, or ACh

Exogenous GLP‐2 administration did not influence (*p* = 0.28) the vasoactive response of the isolated MV to increasing GLP‐2 concentrations ex vivo (Figure 2a). However, vasorelaxation responses were greater at lower GLP‐2 concentrations (5 × 10^−9^ M to 1 × 10^−7^ M) compared with greater GLP‐2 concentrations (1 × 10^−6^ M to 1 × 10^−5^ M) (Figure 2a). There was a s.c. injection × ex vivo VIP concentration interaction (*p* = 0.05) in the isolated MV because the MV from BSA‐treated steers relaxed more than the MV from GLP‐2‐treated steers at 1 × 10^−6^ M, but not with other doses of VIP (Figure 2b). Exogenous GLP‐2 administration did not influence (*p* = 0.44) the vasoactive response to 5‐HT (Figure 2c). Increasing concentrations of 5‐HT (*p* < 0.001) progressively reduced vasorelaxation, with minimal relaxation at 1 × 10^−5^ M and 1 × 10^−4^ M, suggesting a greater contribution of contractile influences at greater concentrations of 5‐HT (Figure 2c). As ACh concentrations increased, vasorelaxation increased in the isolated MV in a dose‐dependent manner (*p* < 0.001) (Figure 2d). Exogenous GLP‐2 administration did not influence (*p* = 0.45) the vasoactive response of isolated MV to ACh. For ‐log EC_50_ values, no differences were observed among s.c. injection treatments (*p* ≥ 0.32; Table 4), except for ACh, for which a trend was detected (*p* < 0.09), with lower values in veins from GLP‐2‐treated animals (4.18) compared with control animals (2.77).

**FIGURE 2:**
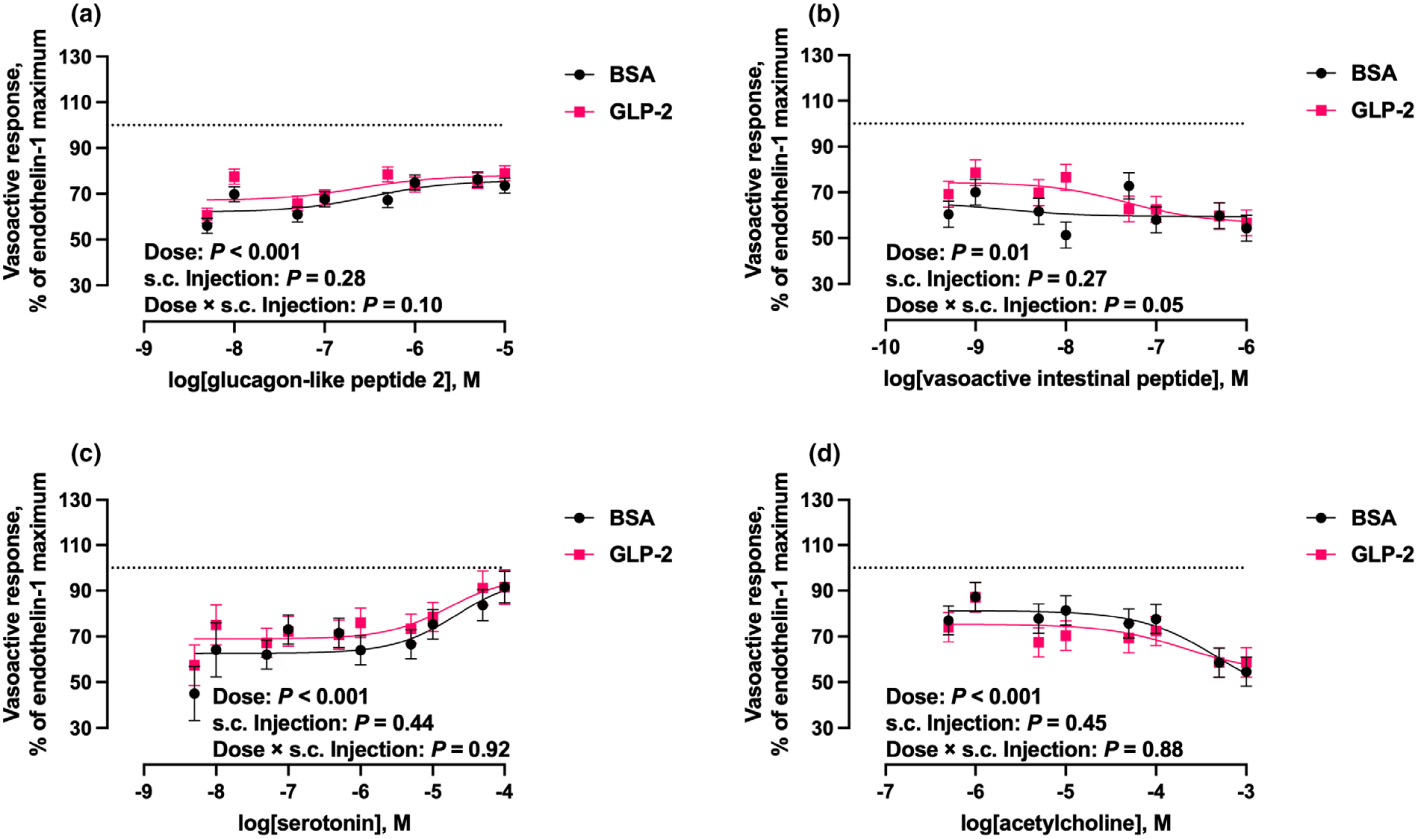
Least squares mean contractile responses (± SEM; *n* = 5) of the mesenteric vein to increasing concentrations of (a) GLP‐2, (b) VIP, (c) 5‐HT, and (d) ACh in steers treated in vivo with bovine serum albumin (BSA; control) or GLP‐2. *p* values: (a) GLP‐2 dose (*p* < 0.001), s.c. injection (*p* = 0.28), and the dose × s.c. injection interaction (*p* = 0.102). (b) VIP dose (*p* = 0.01), s.c. injection (*p* = 0.27), and the dose × s.c. injection interaction (*p* = 0.05). (c) 5‐HT dose (*p* < 0.001), s.c. injection (*p* = 0.44), and the dose × s.c. injection interaction (*p* = 0.92). (d) ACh dose (*p* < 0.001), s.c. injection (*p* = 0.45), and the dose × s.c. injection interaction (*p* = 0.88). The dotted line at 100% represents the maximal contractile response to endothelin‐1.

## 4 DISCUSSION

Exogenous GLP‐2 administration increases small intestinal mass by stimulating crypt cell proliferation, resulting in greater villus height, crypt depth, and overall mucosal mass, while enhancing intestinal barrier function (Burrin et al., 2007; Deng et al., 2016; Taylor‐Edwards et al., 2011; Trotta, Swanson, et al., 2023; Trotta, Swanson, et al., 2024). These intestinotrophic responses have been associated with increased mesenteric blood flow across multiple species, thereby increasing nutrient and oxygen delivery to the mucosa to support intestinal metabolism and function (Galsgaard et al., 2025; Guan et al., 2006; Høyerup et al., 2013; Taylor‐Edwards et al., 2011). Limitations of the present study include a lack of measurements of small intestinal mass or indices of small intestinal growth in response to exogenous GLP‐2; however, similar protocols have resulted in intestinotrophic effects and increased plasma GLP‐2 concentrations (Burrin et al., 2007; Taylor‐Edwards et al., 2011). Although GLP‐2‐induced intestinal growth is thought to be mediated through indirect neuroendocrine pathways, the mechanisms regulating associated vascular responses remain incompletely understood. Accumulating evidence suggests that the signaling pathways responsible for GLP‐2‐induced increases in mesenteric blood flow are species‐dependent (Galsgaard et al., 2025; Guan et al., 2003). Therefore, the objective of this study was to characterize the vascular signaling mechanisms associated with GLP‐2‐mediated increases in mesenteric blood flow in cattle. To our knowledge, this is the first study to directly evaluate vascular effects of GLP‐2 in ruminants using a myography‐based approach.

### 4.1 Influence of GLP‐2 on blood vessels dimensions

To our knowledge, there is little information documenting the effects of exogenous GLP‐2 administration in animals on vascular morphology post‐mortem. Most of the literature has focused on the effects of exogenous GLP‐2 on small intestinal growth, while splanchnic vascular responses to exogenous GLP‐2 have been measured in vivo (Bremholm et al., 2010; Deniz et al., 2007; Guan et al., 2003; Stephens et al., 2006; Taylor‐Edwards et al., 2011). In the current study, exogenous GLP‐2 administration did not alter vascular dimensions of either the MA or MV. In nonruminants, GLP‐2‐mediated increases in mesenteric blood flow occur rapidly, within minutes of administration (Bremholm et al., 2010; Deniz et al., 2007; Guan et al., 2003; Stephens et al., 2006). This rapid increase in blood flow appears to reflect an acute functional vasorelaxation response through transient reductions in vascular resistance rather than structural remodeling of the mesenteric vasculature. Nevertheless, the possibility that prolonged GLP‐2 exposure induces vascular remodeling cannot be excluded, as the dosing of the current study represents an acute exposure to exogenous GLP‐2. Alternatively, prolonged GLP‐2 administration has been suggested to induce GLP‐2 receptor desensitization (Taylor‐Edwards et al., 2011), thereby limiting sustained vascular responses.

### 4.2 Influence of GLP‐2 on GLP‐2‐mediated vasoactivity

Although the mechanisms underlying GLP‐2‐mediated increases in mesenteric blood flow remain unclear, low levels of *GLP2R* mRNA have been identified in murine MA (Yusta et al., 2019), suggesting that direct receptor activation could potentially contribute to vascular regulation. In the current study, *GLP2R* mRNA was detected in both MA and MV of the steers. However, increasing GLP‐2 exposure ex vivo did not result in vascular responses despite detectable *GLP2R* mRNA. These findings suggest that GLP‐2 does not directly induce vasorelaxation through activation of GLP2R on vascular smooth muscle or endothelial cell membranes. However, low GLP2R protein expression or functionality may also explain the lack of response.

In the current study, increasing concentrations of GLP‐2 ex vivo had minimal effects on the vascular relaxation of the isolated bovine MA and MV. The greatest vasorelaxation in response to GLP‐2 ex vivo was observed in the MV at 5 × 10^−9^ M, the lowest dose used in the current study. Plasma GLP‐2 concentrations under normal physiological conditions are typically less than 5 × 10^−11^ M, but can increase to 1.5 × 10^−9^ M with similar exogenous GLP‐2 treatment (Taylor‐Edwards et al., 2011). It is possible that lowering the dose range tested could have yielded different results because of complex ligand‐receptor interactions that are not yet understood. Nonetheless, greater doses would be expected to elicit a response if there was any to be observed. Therefore, the lack of GLP‐2‐mediated vasorelaxation ex vivo supports the hypothesis that GLP‐2‐induced increases in mesenteric blood flow are mediated indirectly through neuroendocrine intermediates. This interpretation is consistent with previous work demonstrating that GLP2R is predominantly localized to enteric neurons and enteroendocrine cells rather than vascular cells (Guan et al., 2006). Similarly, others have reported intestinotrophic effects of exogenous GLP‐2 in rats without changes in *GLP2R* mRNA or protein expression, suggesting that biological responses may depend on activation of pre‐existing GLP2R and downstream signaling pathways, rather than GLP2R upregulation (Ren et al., 2018). Collectively, these results suggest that GLP‐2‐mediated increases in mesenteric blood flow are secondary to downstream signaling pathways rather than direct GLP2R activation in vascular cells.

### 4.3 Influence of GLP‐2 on vasoactive intestinal peptide‐mediated vasoactivity

Vasoactive intestinal peptide is recognized as a potent vasodilator and generally exhibits greater vasodilatory potency than ACh (Fahrenkrug, 1989). Indeed, we observed that VIP was substantially more potent than ACh in both MA and MV relaxation. In the MA, VIP potency exceeded that of ACh by approximately 132‐fold, whereas in the MV the difference exceeded 50,000‐fold based on ‐log EC_50_ comparisons. Dose‐dependent vasorelaxation responses to VIP have been documented in isolated bovine coronary arteries and rat pulmonary artery rings using isometric tension recordings in organ bath systems (Itoh et al., 1990; Zhang et al., 2010). However, to our knowledge, the current study is the first to characterize VIP‐mediated vasorelaxation in isolated bovine MA and MV using myography techniques. In the present study, VIP increased vasorelaxation in a dose‐dependent manner in both the MA and MV. Maximal vasorelaxation occurring in response to VIP was approximately 55% in the MA and 40% in the MV, confirming the potent vasodilatory role of VIP within the mesenteric circulation.

Physiological effects of VIP are mediated through activation of VPAC1 and VPAC2 receptors (*Bos taurus* gene names *VIPR1* and *VIPR2*, respectively). In mice, VPAC2 receptors were found predominantly in smooth muscle, including blood vessels and in the smooth muscle layers of the gastrointestinal and reproductive systems, the basal part of the mucosal epithelium in the colon, lung, the vasculature of the kidney, adrenal medulla, and retina (Harmar et al., 2004). Vasodilatory effects of VIP are mediated via VPAC1 activation on endothelial cells, followed by release of NO, and via VPAC2 activation on vascular smooth muscle cells in the porcine basilar arteries (Grant et al., 2006). In the present study, *VIPR1* mRNA was not detected in either MV or MA, whereas expression of *VIPR2* mRNA was detected in both bovine MA and MV in the current study. Therefore, VIP receptor‐mediated vascular relaxation is likely mediated by *VIPR2* in bovine MA and MV. Direct antagonism of *VIPR2* would be needed to evaluate this hypothesis. It is worth noting that the greater *NOS3* mRNA expression in the MV from GLP‐2‐treated steers represents, to our knowledge, a novel finding in cattle and may suggest a potential role for NO in the regulation of venous tone. However, because *VIPR1* mRNA was not detected in the vessels evaluated and *VIPR2* expression was confirmed, it is more likely that the vasodilatory effects observed in the present study are mediated by direct mechanisms of VIP involving activation of VIPR2 in vascular smooth muscle, independent of NO.

### 4.4 Influence of GLP‐2 on serotonin‐mediated vasoactivity

Serotonin is a complex regulator of vascular tone because activation of different receptor subtypes can result in either vasoconstriction or vasorelaxation (Hoyer et al., 1994; Snider et al., 2018; Trotta, Harmon, & Klotz, 2024). In cattle, activation of HTR2B, HTR4, and HTR7 has been associated with vasorelaxation, whereas HTR4 is the predominant serotonergic receptor mediating relaxation of isolated bovine peripheral veins (Trotta, Harmon, & Klotz, 2024; Trotta & Klotz, 2026). In the present study, exogenous GLP‐2 treatment did not influence vasorelaxation responses to 5‐HT, which corresponded with a lack of changes in serotonergic receptor mRNA expression. Although there were numerical increases in *HTR4* mRNA expression in MA from GLP‐2‐treated animals, this did not translate into measurable functional differences. The limited vasorelaxation response to 5‐HT likely reflects simultaneous activation of vasodilatory and contractile serotonergic pathways, particularly signaling through HTR2A, which is abundantly expressed in MA and mediates vasoconstriction (Kato et al., 1999; Snider et al., 2018). The lack of HTR2A antagonism and the absence of its mRNA evaluation limit further mechanistic interpretation. In addition, 5‐HT may require the release of secondary mediators, such as VIP, to exert vasodilatory effects (Chou, 1989), which may not occur under ex vivo conditions using isolated blood vessels.

Supporting this interpretation, in vivo studies have shown that 5‐HT increases splanchnic blood flow (Seitz & Watts, 2014), whereas ex vivo studies report minimal or no direct vasorelaxation when 5‐HT is applied to isolated arteries, either under basal or pre‐contracted conditions (Davis et al., 2012). 5‐hydroxytryptamine receptor 2A is considered the predominant serotonergic receptor mediating vascular contraction in gastrointestinal vessels of cattle (Snider et al., 2018; Trotta, Harmon, & Klotz, 2024). Increasing concentrations of 5‐HT dose‐dependently increased vasoconstriction in the MV, suggesting a greater contribution of contractile mechanisms at greater concentrations, consistent with previous studies (Trotta, Harmon, et al., 2023; Trotta, Harmon, & Klotz, 2024). Overall, the vascular response to 5‐HT appears to depend on multiple factors, including species, vessel type, and the heterogeneous distribution of receptor subtypes in vascular tissues (Ni & Watts, 2006; Snider et al., 2018).

### 4.5 Influence of GLP‐2 on acetylcholine‐mediated vasoactivity

Acetylcholine is a well‐established vasodilator that stimulates endothelial muscarinic receptor M3 (CHRM3) signaling, thereby increasing intracellular Ca^2+^ concentrations and activating endothelial nitric oxide synthase (eNOS) through the canonical NO‐soluble guanylate cyclase‐cGMP pathway (Furchgott & Zawadzki, 1980; Sonobe & Kakinuma, 2024). In addition to NO‐dependent mechanisms, ACh‐mediated vasorelaxation can also occur through NO‐independent pathways (Jarrett et al., 2013; Saternos et al., 2025; Surprenant, 1994; Venkatesan et al., 2026). Consequently, ACh induces relaxation in both conduit and resistance vessels across species (Lüscher et al., 1987; Vanhoutte & Miller, 1985).

In the current study, steers treated with exogenous GLP‐2 exhibited greater vasorelaxation in the MA at the greatest ex vivo ACh doses. These findings suggest that the vascular effects of GLP‐2 are primarily functional rather than structural and may involve enhanced endothelial responsiveness to cholinergic stimulation. Although *CHRM3* mRNA expression was not significantly altered, numerical increases in GLP‐2‐treated MA provide a plausible mechanistic basis for enhanced ACh responsiveness observed ex vivo. Direct mechanistic links between GLP‐2 signaling and vascular cholinergic pathways remain mostly uncharacterized. However, pharmacological activation of CHRM3 with a selective agonist was shown to induce vasodilation and reduce blood pressure in experimental models of pulmonary hypertension (Ahmed et al., 2016) and muscarinic receptors are recognized regulators of endothelial NO release (Alves‐Lopes et al., 2019). The CHRM3 is expressed in both endothelial cells and vascular smooth muscle cells, resulting in context‐dependent physiological responses. In the presence of intact endothelium, CHRM3 activation primarily induces vasodilation through NO signaling, whereas activation of CHRM3 on smooth muscle cells in the absence of functional endothelium can promote vasoconstriction (Dauphin & Hamel, 1990; Gericke et al., 2014; Harvey, 2012).

Interestingly, Galsgaard et al. (2025) reported that GLP‐2‐induced increases in mesenteric blood flow in mice were independent of both NO and VIP signaling, although cholinergic pathways were not evaluated. Based on the enhanced ACh responsiveness observed in the MA, cholinergic signaling may represent a previously unrecognized contributor to GLP‐2‐associated mesenteric vasoregulation in cattle. Future studies should determine whether these responses involve cGMP‐dependent signaling pathways or alternative endothelial mechanisms.

## 5 CONCLUSION

To our knowledge, this is the first study to use a myography‐based approach to investigate the vascular mechanisms associated with GLP‐2‐induced mesenteric blood flow responses in cattle. Exogenous GLP‐2 administration did not directly induce vasorelaxation in isolated MA or MV despite detectable *GLP2R* mRNA expression, indicating that GLP‐2‐mediated mesenteric vasodilation likely occurs through indirect neuroendocrine signaling pathways rather than direct receptor activation within vascular tissue. Furthermore, exogenous GLP‐2 administration did not alter mesenteric vascular dimensions but increased *NOS3* mRNA expression in the MV and enhanced ACh‐mediated vasorelaxation in the MA at greater agonist concentrations. Collectively, these findings suggest that cholinergic signaling pathways may contribute to GLP‐2‐associated mesenteric vascular regulation in cattle.

## AUTHOR CONTRIBUTIONS

**Gabriel S. S. David:** Formal analysis; visualization; writing – original draft; writing – review and editing. **Erica C. Maples:** Formal analysis; investigation; visualization; writing – original draft; writing – review and editing. **James L. Klotz:** Conceptualization; investigation; methodology; project administration; resources; writing – review and editing. **Ronald J. Trotta:** Conceptualization; formal analysis; investigation; methodology; project administration; resources; supervision; writing – original draft; writing – review and editing.

## FUNDING INFORMATION

This research was not supported by any particular source of funding.

## CONFLICT OF INTEREST STATEMENT

There are no real or perceived conflicts of interest.

## ETHICS STATEMENT

All animal procedures were approved by the University of Kentucky Institutional Animal Care and Use Committee (Protocol No. 2020‐3479).

## DISCLOSURES

Mention of trade name, proprietary product of specified equipment does not constitute a guarantee or warranty by the USDA and does not imply approval to the exclusion of other products that may be available.

## Data Availability

Data will be made available upon reasonable request to the corresponding author.
